# Imaging-based frequency mapping for cochlear implants – Evaluated using a daily randomized controlled trial

**DOI:** 10.3389/fnins.2023.1119933

**Published:** 2023-04-13

**Authors:** Lars Lambriks, Marc van Hoof, Joke Debruyne, Miranda Janssen, Josef Chalupper, Kiki van der Heijden, Janny Hof, Katja Hellingman, Elke Devocht, Erwin George

**Affiliations:** ^1^Department of ENT/Audiology, School for Mental Health and Neuroscience, Maastricht University Medical Centre, Maastricht, Netherlands; ^2^Department of Methodology and Statistics, Care and Public Health Research Institute, Maastricht University, Maastricht, Netherlands; ^3^Advanced Bionics European Research Centre, Hannover, Germany

**Keywords:** cochlear implant, imaging-based fitting, tonotopy, frequency allocation table (FAT), pitch mismatch, daily randomization, randomized controlled trial (RCT), cone beam CT

## Abstract

**Background:**

Due to variation in electrode design, insertion depth and cochlear morphology, patients with a cochlear implant (CI) often have to adapt to a substantial mismatch between the characteristic response frequencies of cochlear neurons and the stimulus frequencies assigned to electrode contacts. We introduce an imaging-based fitting intervention, which aimed to reduce frequency-to-place mismatch by aligning frequency mapping with the tonotopic position of electrodes. Results were evaluated in a novel trial set-up where subjects crossed over between intervention and control using a daily within-patient randomized approach, immediately from the start of CI rehabilitation.

**Methods:**

Fourteen adult participants were included in this single-blinded, daily randomized clinical trial. Based on a fusion of pre-operative imaging and a post-operative cone beam CT scan (CBCT), mapping of electrical input was aligned to natural place-pitch arrangement in the individual cochlea. That is, adjustments to the CI’s frequency allocation table were made so electrical stimulation of frequencies matched as closely as possible with corresponding acoustic locations in the cochlea. For a period of three months, starting at first fit, a scheme was implemented whereby the blinded subject crossed over between the experimental and standard fitting program using a daily randomized wearing schedule, and thus effectively acted as their own control. Speech outcomes (such as speech intelligibility in quiet and noise, sound quality and listening effort) were measured with both settings throughout the study period.

**Results:**

On a group level, standard fitting obtained subject preference and showed superior results in all outcome measures. In contrast, two out of fourteen subjects preferred the imaging-based fitting and correspondingly had better speech understanding with this setting compared to standard fitting.

**Conclusion:**

On average, cochlear implant fitting based on individual tonotopy did not elicit higher speech intelligibility but variability in individual results strengthen the potential for individualized frequency fitting. The novel trial design proved to be a suitable method for evaluation of experimental interventions in a prospective trial setup with cochlear implants.

## 1. Introduction

Cochlear implants make use of the tonotopic organization of the cochlea by assigning low frequency sounds to apical electrodes and high frequency sounds to basal electrode contacts. In current clinical practice, a default frequency allocation table (FAT) is commonly assigned, which ignores the precise location of electrodes along the cochlea. There is, however, substantial variation in cochlear morphology and electrode positioning across individuals (Finley and Skinner, 2008; Meng et al., 2016). As a result, a standardized FAT generally induces a substantial mismatch between the acoustic input frequency and the natural tonotopic frequency of the spiral ganglion cells targeted by the electrode contact. Indeed, literature reports substantial frequency-to-place mismatch in cochlear implant (CI) users (Landsberger et al., 2015; Canfarotta et al., 2020; Mertens et al., 2022). Therefore, we investigated in a prospective, controlled, randomized and single-blinded setting if reducing this mismatch in individual patients leads to an improvement in CI performance.

During rehabilitation, post-lingual CI patients typically acquire the greatest improvements in speech understanding during the first months, and then improve with smaller increments on the long term (Holden et al., 2013; Kelsall et al., 2021). It is assumed that patients who were familiarized with a normal frequency-to-place function before hearing loss, need to acclimatize to the frequency shifted signals provided by the CI. To date, it is unclear how this mismatch affects clinical outcomes. Recent studies have reported a significant correlation between frequency shift and speech perception in subjects implanted with a MED-EL electrode array (Canfarotta et al., 2020; Mertens et al., 2022). Here, speech performance was better in subjects with a smaller frequency-to-place mismatch. Although in Mertens et al. (2022) mismatch significantly correlated with speech perception at 6 months, this effect disappeared 12 months after CI activation. This is in line with the hypothesis that the human brain is able to adapt to a tonotopic frequency shift over time. It is likely however, that this adaptation process prolongs the rehabilitation period. There seems to be a high variability in CI subjects’ ability to adapt to this pitch mismatch (Reiss et al., 2014; Tan et al., 2017), leaving some users with incomplete adaptation even after long periods of time. Possibly, auditory plasticity is able to overcome smaller amounts of mismatch but cannot always compensate for greater degrees of mismatch (Svirsky et al., 2015).

One strategy to minimize frequency-to-place mismatch and diminish the need for adaptation is to align frequency assignment in the CI processor with individual electrode positioning. Previously, Grasmeder et al. (2014) found that frequency maps based on estimated insertion angles did not improve speech intelligibility compared to standard maps. Fu et al. (2002) did not measure electrode positioning directly but applied an apical shift of 2–4 mm in frequency assignment relative to clinical settings. Speech recognition deteriorated with the experimental settings, despite a 3 month habituation period. Recently, a pilot study was performed in which implanted subjects showed better results on speech discrimination tests with an anatomically based FAT compared to their routine clinical settings (Di Maro et al., 2022). Other strategies to improve CI frequency distributions have also been evaluated, such as evolutionary algorithms and smartphone applications which enable FAT self-adjustment by patients (Fu et al., 2002; Jethanamest et al., 2017; Saadoun et al., 2022).

Besides the limited number of experimental studies on FAT optimization also some methodological issues arise that have to be taken into account. Most CI studies selected experienced CI patients who already adapted to a clinical FAT setting. This increases the risk of a first-order carryover effect in which there is a bias toward any setting that is given first during the initial rehabilitation period due to the extensive neural plasticity following CI implantation (Middlebrooks et al., 2005; Fallon et al., 2008; Strelnikov et al., 2015). For example, it is conceivable that CI users who receive setting A followed by setting B will generally favor setting A as a result of initial post-implantation brain plasticity rather than as a result of the beneficial properties of setting A. This bias restricts the use of a conventional crossover trial in which subjects are exposed to settings in sequential order and effectively requires a parallel test-versus-control setup. A Randomized Controlled Trial (RCT) (in which subjects are assigned to either setting A or B) would be the preferred design to overcome this issue, but requires the double number of participants to achieve reasonable statistical power, and also increases the risks of suboptimal treatment in one of the two study groups. Moreover, between-patient factors such as implant type, duration of deafness and age of implantation have been reported to impact CI performance and thus may decrease group comparability and validity of results, especially with the availability of small sample sizes (Gaylor et al., 2013; Beyea et al., 2016).

To the best of our knowledge, this current study is the first controlled attempt to provide imaging-based frequency mapping to CI patients, immediately from the start of rehabilitation. We present the results of the ELEPHANT (ELEctrically Place-pitched Hearing Achieves Natural Tonotopy) study, a clinical trial in which an imaging-based fitting intervention is evaluated using an alternative type of trial design (Lambriks et al., 2020). Here, frequency-to-place mismatch was reduced by aligning electrical input to natural place-pitch arrangement of the individual cochlea. To prevent carryover effects, subjects stood as their own control and treatment allocation was instead based on daily randomization. The aim of this study was to investigate the difference between imaging-based and standard fitting on outcomes that relate to speech perception and sound quality. It was hypothesized that the imaging-based fitting strategy would give rise to a steeper learning curve (less adaptation needed) and would result in a more favorable outcome (better speech recognition) as electrical stimulation is matched to the natural tonotopy of a normal hearing human brain. Also, the intervention was expected to restore natural sound quality and win subject preference.

## 2. Materials and methods

### 2.1. Ethical approval

This study has been approved by the ethics committee of the Maastricht University Medical Center (MUMC+) and has been registered in the Clinical Trials Register (NL64874.068.18) and at ClinicalTrials.gov (NCT03892941). The study was conducted in compliance with the Declaration of Helsinki and ISO 14155:2020 (Clinical investigation of medical devices for human subjects - Good clinical practice). Study outcomes were reported according to the CONSORT guidelines (Schulz et al., 2010). Subjects provided informed consent before participation and received compensation only for their travelling costs that were due to study participation.

### 2.2. Design

This trial was designed by the sponsor (MUMC+) to be a single-blinded, controlled clinical study with daily crossover randomization. Subjects were recruited prior to surgery and treatment exposure was implemented directly from the start of CI rehabilitation. A detailed description of the trial set-up, divided in three different phases, is given in its protocol publication (Lambriks et al., 2020). In the current manuscript, the results of phase 1 are reported. In short, a new type of trial design was implemented where subjects acted as their own control and treatment allocation was based on daily randomization. For a period of three months, starting at first fit, subjects followed a randomization scheme whereby each individual crossed over between the interventional and standard fitting program. Here, two speech processors (physically labeled with either a circle or a triangle) were distributed, one of which was programmed with the intervention settings and the other with the standard settings. Each day, subjects were allocated to wear one of both processors. The randomization period was followed by a 3-month period (in between regular clinical visits at 3 and 6 months postoperatively) of free choice in which patients had the liberty of choosing whatever program they preferred.

The within-subject randomization of the wearing schedule was performed using a customized script in Wolfram Mathematica 12.0 (Wolfram Research, Champaign, IL, USA) in a 1:1 ratio between both fittings. To prevent subjects from developing a preference for one of both programs due to unequal exposure during the first crucial period of CI adaptation, several prerequisites were included in the randomization procedure. First, the same processor was not allocated for more than two consecutive days within the first four weeks of CI rehabilitation. From four weeks onward, this restriction was loosened to a maximum of four consecutive wearing days. Second, to aid subject blinding and in order to prevent subjects to habitually prefer one processor over the other, the assignment of the two fittings across the two processors was randomized by the fitting clinician according to a schedule. As such, there was a 50% chance at each fitting that the imaging-based program was saved on the processor with either the circle or the triangle. Data collection and study visits for the study phase presented in this paper followed the schedule in Table 1. In this manuscript, time points are referred to as the number of weeks after first CI activation (for example, +4 weeks).

**TABLE 1:** Schedule of enrollments and measurements where timepoint 0 represents CI activation (+0 weeks).

		Weeks after CI activation													
**Timepoint**	**Description**	**#**	**−4**	**−3**	**0**	**+1**	**+2**	**+3**	**+4**	**+5**	**+6**	**+7**	**+8**	**+10**	**+12**
**Enrollment**															
Clinical pre-assessment	CI screening selection	X													
Informed consent		X													
Clinical CT/MRI		X													
Surgery			X												
CBCT scan				X											
**Primary outcomes**															
Patient preference	10-point VAS scale				X	X	X	X	X	X	X	X	X	X	X
Word recognition	CNC	X			X	X	X	X	X	X	X	X	X	X	X
Sentence recognition in quiet	Dutch Matrix Sentence Test				X	X	X	X	X	X	X	X	X	X	X
Sentence recognition in noise	Dutch Matrix Sentence Test				X	X	X	X	X	X	X	X	X	X	X
**Secondary outcomes**															
Listening effort	13-point VAS scale								X						X
Loudness scaling	ACALOS								X						X
Frequency selectivity	SMRT								X						X
Sound quality	Questionnaire							X							X

Primary outcomes were measured at every visit during rehabilitation and secondary outcomes only at specified visits.

### 2.3. Study population

Inclusion took place in a tertiary care university medical center in the Netherlands (MUMC+, Maastricht, Netherlands). Adult subjects (>18 years of age) who were Dutch speaking, eligible for receiving a CI (according to the Dutch implantation criteria), opted to receive a HIRes Ultra implant with HiFocus Midscala electrode array (Advanced Bionics, Sylmar, CA, USA) and were willing to participate in the study, were eligible for inclusion. Exclusion criteria were contraindications for CT imaging, cochlear or neural abnormalities that could compromise the placement of the electrode or affect outcome measures, opportunities for electric-acoustic stimulation (EAS) within the first year follow-up, previous or bilateral implantation, early onset of profound deafness (<4 years of age), and additional disabilities that could prevent active trial participation.

### 2.4. Study intervention

Fitting of subjects was performed with research processors (Naída Q70) using research software BEPS+. To reach the full potential for each setting, maps were fitted separately, with real-life adjustments based on behavioral M and T levels, as normally done in clinical routine using Soundwave™. Differences in M and T levels between both maps were kept within 10 Clinical Units (CU). In both maps, the HiRes Optima S processing strategy was used, Input Dynamic Range (IDR) was set to 60 dB, ClearVoice was set to medium, and all other preprocessing features (such as SoftVoice) were disabled. Fitting sessions were performed by two experienced clinical audiologists. Since BEPS+ did not support data logging, subjects were asked to report the wearing time of their CI processors in daily diaries. By comparing participant reported wearing time with intended wearing time according to the randomization schedule, compliance differences were calculated and compared to predefined cut-off points to determine study continuation (Lambriks et al., 2020).

In the interventional program, frequency mapping of the CI was applied as such that frequency distribution (FAT) across the electrode array matched the corresponding tonotopic locations as closely as possible. The details of the procedure are explained in Lambriks et al. (2020). In short, a postoperative Cone Beam CT (CBCT) scan was acquired one week after surgical placement of the cochlear implant. This scan was fused (Dees et al., 2016) with the available preoperative imaging (CT/MRI) using 3D Slicer (Fedorov et al., 2012) and BRAINSFit software (Johnson et al., 2007) after which intra-cochlear electrode positioning was assessed by placing markers at the center of each contact, as validated before (Devocht et al., 2016). The lateral wall was marked manually starting at the round window up to the helicotrema, at a height corresponding to the basilar membrane. The insertion position was calculated for each electrode by estimating the distance in millimeters from the round window to the nearest point on the lateral wall for each contact. Tonotopic electrode frequency was calculated by applying the Greenwood function to the insertion depth relative to the subject’s cochlear duct length (Greenwood, 1990).

The calculated tonotopic locations of electrodes were used to create a tonotopic FAT, which pursued individualized tonotopic alignment. Here, electrode tonotopic frequencies were allocated to the lower bounds of corresponding electrode channels in the CI fitting software. With imaging-based mapping, theoretically, each electrode stimulates those frequency inputs that align with its tonotopical location. Full tonotopical alignment is however not always feasible since strict matching of frequency information would result in loss of low-frequency information that is important for speech perception. Also, on the basal portion of the electrode there are likely to be contacts that fall out of the frequency range covered by a CI processor. To address these issues, the following set of rules was applied: a minimum of two channels stimulated below 1,000 Hz, four channels below 2,000 Hz, seven channels below 4,000 Hz, and the most basal channel had to be stimulated by 8,598 Hz or lower. Also, in the imaging-based FAT, the Advanced Bionics Phantom functionality (further referred to as virtual channel) was enabled to deliver low-frequency information beyond the most apical electrode of the array, providing more flexibility for tonotopic mapping (Saoji and Litvak, 2010). The virtual channel was included as a channel in the rule set specified above. Results of imaging and relationships with electrophysiological outcomes are elaborated upon in a separate publication (Lambriks et al., 2023).

### 2.5. Primary outcomes

Measurements were performed in a sound-attenuated booth. The order of outcome measurements was fixed, where sessions started with preference scales and CNC word recognition, followed by sentence recognition in quiet and noise, and if applicable, listening effort, loudness scaling, and frequency selectivity (Table 1). Measurements were performed in the CI only condition with both the imaging-based and standard fitting in a randomized order. Testing procedures are explained in detail in Lambriks et al. (2020), and in short in the following sections.

#### 2.5.1. Preference scales

To determine subject preference in everyday life, subjects were asked to rate their satisfaction with both settings in a short questionnaire at every visit. Satisfaction was rated on a 10-point Visual Analogue Scale (VAS) with respect to speech understanding, sound quality and sound recognition.

#### 2.5.2. CNC word recognition

Word recognition was evaluated with phoneme scoring at the level of 65 dB SPL with two lists (test-retest) on a Dutch monosyllabic consonant-nucleus-consonant (CNC) speech recognition test (Bosman and Smoorenburg, 1995). The average of test-retest values was calculated as the primary outcome value.

#### 2.5.3. Sentence recognition

The Dutch Matrix Sentence test (Theelen–van den Hoek et al., 2014; Houben and Dreschler, 2015) with 10 sentence lists (test-retest) was used to measure sentence recognition both in quiet and in background noise using the Oldenburg Measurement Applications (OMA) software (HörTech gGmbH, Oldenburg, Germany). Subjects were asked to reconstruct sentences by selecting perceived words from a closed set using a touch screen. In quiet, the percentage of correct answers was recorded at a speech level of 65 dB SPL. In noise, an adaptive procedure determined the Speech Reception Threshold (SRT), which is defined as the signal-to-noise ratio (SNR) corresponding to a 50% correct score. Here, noise was fixed at a level of 65 dB SPL while speech level was varied according to scoring performance. Scores were *post-hoc* excluded from analysis when the procedure evoked an invalid SRT outcome. This was defined as scores higher than 15 dB SNR, or if the resulting SNR was outside the range of presented levels (Kaandorp et al., 2015). In some cases, sentence recognition in quiet was not measured when judged as too difficult after CNC score evaluation. Then, a score of 20% was recorded to correct for guessing. Sentence recognition in noise was only measured if the score in quiet was higher than or equal to 50%, otherwise an SRT value of 15 dB SNR was assigned.

### 2.6. Secondary outcomes

#### 2.6.1. Listening effort

Listening effort was evaluated using the same set of sentences from the Dutch Matrix Sentence test as used in the speech test. Here, subjects rated the amount of effort required to listen to the presented speech in noise. During this procedure, subjective rating is monitored using a 13-point scale which ranges from no effort to extreme effort on a touch screen. The noise level was fixed at 65 dB SPL, while speech level was varied in order to create different signal-to-noise ratios according to the individual SRT of each subject, as determined in the corresponding listening condition during sentence intelligibility in noise. Overall, six levels were set around the subjects’ individual SRT (SRT -6, SRT -3, SRT, SRT + 3, SRT + 6, SRT + 9 dB). It should be noted that the test specific SRT levels that were used to set the individual listening effort levels, were set at hoc and thus not yet corrected for the *post hoc* exclusion principles stated in section “2.5.3. Sentence recognition”.

#### 2.6.2. Sound quality

The quality of speech perception was measured with the sound quality questionnaire by Devocht et al. (2017) which is based on the descriptives of Boretzki (1999). Here, subjects rated the applicability of 10 sound quality features on a 10-point VAS scale.

#### 2.6.3. Loudness scaling

Loudness growth was measured with the Adaptive Categorical Loudness Scaling (ACALOS) procedure using Oldenburg Measurement Applications software. Subjects were presented with 1/3-octave band noises (center frequencies 250, 500, 1,000, 2,000, and 4,000 Hz) at different loudness levels. After each stimulus, subjects were asked to rate loudness on an 11-point scale ranging from inaudible to too loud on a touch screen. Loudness growth curves were visualized in a colored display according to a procedure published in another manuscript by Lambriks et al. (2022). To quantify loudness perception, Area Under the Curves (AUC) were calculated across frequencies and for each frequency separately. Results were compared between fitting maps to investigate the effect of frequency distribution on loudness growth.

#### 2.6.4. Frequency selectivity

The spectral-temporally modulated ripple test (SMRT) was used to measure the capability to spectrally resolve frequency information. This adaptive forced-choice test measured the participant’s ability to discriminate stimuli that were modulated in the frequency domain (Aronoff and Landsberger, 2013). During the test, participants were presented with two intervals: one contained a reference stimulus with 20 ripples per octave, the other contained the target stimulus, which had a varying ripple rate. The target stimulus initially had 0.5 ripples per octave, but was modified using a 1-up/1-down procedure with a step size of 0.2 ripples per octave, until the subject could no longer distinguish between reference and target stimulus. Results of the SMRT test will be reported as Supplementary Information.

#### 2.6.5. Questionnaires

The Speech-Spatial-Qualities of Hearing Scale (SSQ-12) (Gatehouse and Noble, 2004; Noble et al., 2013), the Health Utility Index Mark 3 (HUI-3), and the translated Icepop Capability measure for Adults questionnaire (ICECAP-O) (Al-Janabi et al., 2012; van Hoof et al., 2016) were administered. Results of these questionnaires will be published in a separate manuscript.

### 2.7. Safety management

Adverse events, either related to the intervention or not, were prospectively collected. Serious adverse events were reported to the ethics committee as per local requirements.

### 2.8. Study management

The trial was monitored by a monitor (Clinical Trial Center Maastricht, Maastricht, Germany) contracted by the sponsor (MUMC+, Maastricht, Germany). Statistical analyses were performed after data lock. The first manuscript draft was written by the first author and edited by all co-authors. All authors vouch for the fidelity of the study to the protocol and supported the decision to submit the manuscript for publication.

### 2.9. Sample size calculation

Based on the annual number of patients that are selected for a CI of the brand Advanced Bionics within our clinic, it was estimated before study start that a sample size within the range of 20–30 patients would be feasible to include within the study period (18-24 months). Using the concept of effect size as interpreted by Cohen’s d (Cohen, 1992), it was calculated that with the minimum sample size of 20 and a type 1 error of 0.05, an effect size of 0.66 could be detected. With 30 included subjects and the same alpha level, the effect size was 0.53.

### 2.10. Statistical analysis

Mathematica 13.0 (Wolfram Research, Champaign, IL, USA) was used for analysis and visualization of data. Analyses were primarily performed on the intention-to-treat (ITT) population. The per-protocol (PP) population was analyzed separately for primary outcomes. The ITT population consisted of all included subjects and the PP population was defined as the subgroup of subjects who fully completed the study, complied to the randomization procedures and did not show major protocol deviations. Throughout the manuscript, results are presented for the ITT population, and, as specifically indicated, in case of primary outcome measures also for the PP population. Normality was checked with the Shapiro–Wilk test and visual inspection of the outcome distributions. Means with confidence intervals (95%) and medians with confidence intervals (95%) and interquartile ranges (IQR) were presented as descriptives. Given the sample size and *post-hoc* observation of non-normal distributions according to Shapiro–Wilk test, the non-parametric Wilcoxon signed-rank test was used to calculate differences between results with both CI settings. For those outcomes that were measured at every visit (primary outcomes), learning curves were established by applying linear interpolation between visits. Missing values were also handled by linear interpolation. To evaluate differences between both CI settings, learning curves were analyzed with the following parameters: Begin (result at first fitting), End (result after 3 months), AUC (Area Under the Curve, defined as the area between curve and axis during the first 3 months) and Learning rate (rate of learning within the first month; shown for CNC only). Secondary outcomes were analyzed for each visit separately. Here, missing values were not replaced. Outcome measures which were measured at multiple time points or included multiple domains were Holm–Bonferroni corrected for multiple testing. For learning curves, correction was applied for parameters Begin and End. Adjustments for multiple testing were also applied for secondary outcomes listening effort, sound quality and ACALOS.

## 3. Results

### 3.1. Subjects

**FIGURE 1:**
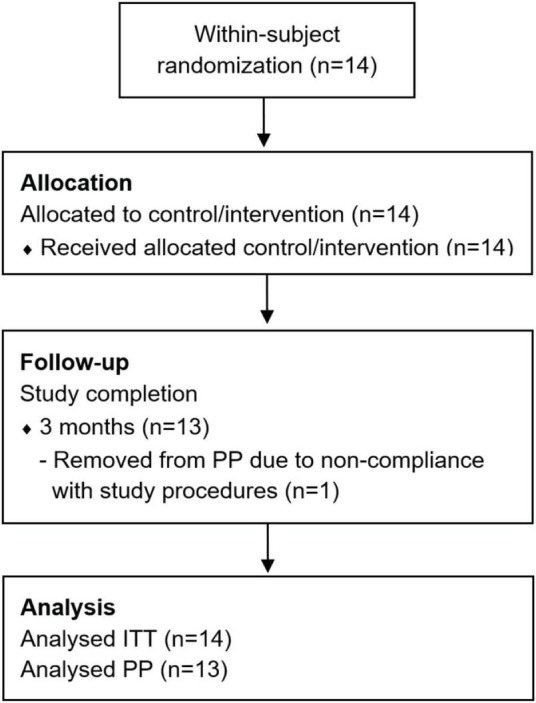
Allocation, follow-up and analysis of subjects during the study.

Fourteen adult patients (eleven male and three female, median age: 65 years, IQR: 9 years) were enrolled in the clinical trial during a recruitment period of 2 years (Figure 1). All participants were Dutch speaking and unilaterally implanted with a HIRes Ultra implant and HiFocus Midscala electrode in the MUMC+. The minimum sample size of 20 subjects, established in the *a priori* sample size calculation, was not reached. Due to the COVID-19 pandemic and subsequent national and local hospital regulations, study inclusion was terminated after the inclusion of the fourteenth patient. It was expected that study deadlines would be threatened if it was decided to wait for mitigation of COVID regulations and for inclusion to re-open. At the time of deciding upon early termination, an interim analysis was performed which showed that sufficient data was already collected to answer the main study goals. Eventually, thirteen subjects (93% of ITT) met the criteria to be included in the PP population. Subject EP07 was excluded due to severe non-compliance with the CI wearing schedule. Baseline characteristics of the ITT population are shown in Table 2. No serious adverse events related to the study intervention were observed.

**TABLE 2:** Subject characteristics.

					PTA (dB HL)		CNC			Etiology (bilateral)		
**Subject**	**Implanted side**	**Age at implantation (years)**	**Duration of hearing loss (years)**	**Onset hearing loss**	**CI ear**	**Contra**	**CI**	**Contra**	**Population**	**Type of loss**	**Course of loss**	**Cause of loss**
EP01	R	64	15	AO	92	107	33%	NHA	PP	SN	Progressive	Unknown
EP02	R	67	12	AO	125	67	NHA	84%	PP	SN	Episodic	Unknown
EP03	L	62	14	AO	117	125	NHA	NHA	PP	SN	Episodic	COM
EP04	L	61	30	AO	73	75	45%	57%	PP	SN	Progressive	Suspected autoimmune
EP05	R	78	29	JO	110	80	NHA	84%	PP	SN	Episodic	COM/LL after bilateral RM
EP06	L	54	24	AO	105	65	78%	60%	PP	SN	Progressive	Unknown
EP07	R	62	26	AO	105	78	NHA	42%	ITT	SN	Progressive	Unknown
EP08	R	78	23	AO	58	73	61%	67%	PP	SN	Sudden	Labyrintitis
EP09	R	64	31	AO	87	80	42%	61%	PP	SN	Progressive	Unknown
EP10	L	78	14	AO	82	77	52%	55%	PP	SN	Progressive	Unknown
EP11	R	60	11	JO	93	83	NHA	79%	PP	SN	Progressive	COM
EP12	R	71	39	JO	120	83	NHA	58%	PP	SN	Progressive	COM
EP13	R	65	39	AO	95	83	30%	60%	PP	SN	Progressive	Suspected hereditary
EP14	R	70	31	AO	70	63	45%	94%	PP	M	Progressive	Otosclerosis/hereditary
Median	71% R	65	25	79% AO	94	79	45%	61%	93% PP	93% SN	–	–
IQR	–	9	17	–	26	10	15%	23%	–	–	–	–

Duration of hearing loss is defined as the number of years from onset of self-reported hearing aid use in the implanted ear until cochlear implant activation. PTA (pure-tone average at 500, 1,000, and 2,000 Hz) is measured preoperatively for the CI ear and for the contralateral ear. CNC scores represent best aided results before surgery (across 55-65-75dB SPL). R, right; L, left; AO, adult onset (>18 years); JO, juvenile onset (>4 years); NHA, no hearing aid; ITT, intention-to-treat, PP, per protocol; IQR, interquartile range; SN, sensori-neural; M, mixed; COM, chronic otitis media; RM, radical mastoidectomy; LL, liquor leakage.

### 3.2. Compliance, protocol deviations and missing values

Supplementary Table 1 shows subject compliance to the randomization procedures expressed as the difference between intended (wearing schedule) and actual (self-reported) wearing (calculated from diaries) of the imaging-based fitting map. Median deviation from subjects’ wearing schedules was 2.4% (IQR 3.9%). Note that these numbers resemble the total exposure during the randomization period, while day-to-day compliance might differ. Other protocol deviations were categorized as deviations from time window (*n* = 17% = 11), missed visits (*n* = 17% = 11; due to national COVID-19 regulations) and missing data (mentioned below). Another protocol deviation that occurred was the misconfiguration (human error, corrected upon discovery) of FATs in two subjects. In EP04, the imaging-based fitting accidentally contained 14 active channels instead of 13 between CI activation and +12 weeks, with the additional channel (electrodes 13–14) having a small (±250 Hz) frequency range which completely overlapped with the range of neighboring channel 13 (electrodes 12–13). In EP09, channel 12 (electrodes 11–12) was set to 5,404–8,530 Hz instead of 5,404–6,491 Hz between CI activation and +3 weeks, thereby overlapping other channels. Protocol deviations were marked as major when compliance regulations to the randomization procedures were violated (Lambriks et al., 2020) or when more than 70% of CNC scores were not measured (*n* = 0). The number of missing values in primary outcomes for imaging-based and standard fitting respectively included 11.2/10.5% for CNC, 2.1/2.1% for preference scales, 11.6/13.7% for sentence recognition in quiet, and 12.3/12.3% for sentence recognition in noise. Listening effort was not measured in 21 and 29% of cases for imaging-based and standard fitting respectively. Loudness scaling was not measured in 14% of cases. The distribution of missing values for the main outcome measure (CNC word recognition), was as such that 11 subjects had no missing measurements, one subject missed 18% of measurements, and two subjects missed 64%. All of these missing values were due to missed visits because of COVID-19.

### 3.3. Imaging-based fitting

#### 3.3.1. Frequency allocation tables

**FIGURE 2:**
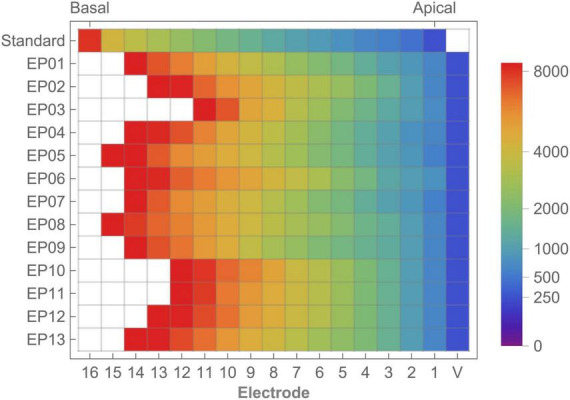
Standard frequency allocation table and individual imaging-based frequency distribution showing the lower frequency bounds for each electrode. Color coding shows frequency allocation for each electrode. White cells indicate disabled electrodes. V, virtual channel.

Data on cochlear morphology and electrode positioning are published separately (Lambriks et al., 2023). Individual filterbanks for imaging-based (different between subjects) and default fitting (fixed) are shown in Figure 2 with color coding and in Supplementary Table 2 with discrete values. In general, imaging-based fitting yielded less (but wider) bands in the low frequencies and more (smaller) bands in the high frequencies (Supplementary Figure 1). Disabling of electrodes in the basal region (due to being outside of the CI frequency range) was required in all subjects for EL16, in 11 subjects for EL15, in 5 subjects for EL14 and in 1 subject for EL13. No electrodes were disabled in the standard fitting.

#### 3.3.2. Frequency-to-place mismatch

**FIGURE 3:**
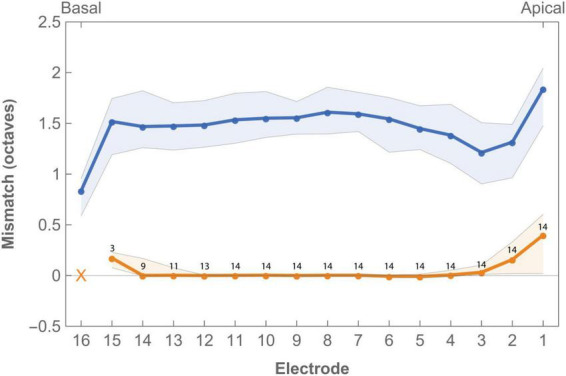
Median frequency-to-place mismatch in octaves across the electrode array for imaging-based (orange) and standard (blue) fitting. Digits indicate the number of subjects in which the electrode was enabled in imaging-based fitting. Electrode 16 was disabled in all imaging-based maps as tonotopic location of this contact was always greater than the highest frequency covered by the CI. Virtual channel not included in graph. Error bands indicate first and third quartile.

Tonotopic mismatch of the standard FAT compared to calculated Greenwood frequency position varied across subjects and electrodes (Figure 3 and Supplementay Table 3). Median mismatch across the array was 1.50 octaves (IQR 0.14) with the most apical electrode showing the highest mismatch (1.84 octaves, IQR 0.57) while the lowest mismatch was found for the basal electrode (0.84 octaves, IQR 0.37). Frequency-to-place mismatch significantly decreased after imaging-based fitting.

### 3.4. Primary outcomes

#### 3.4.1. FAT choice

After 3 months of randomization and 3 months of free choice, two subjects (EP03 and EP12) chose to continue CI use with the imaging-based fitting and 12 subjects retained the standard fitting (14.3% chose intervention, CI^95%^ 0.02–0.45). In the PP population, 15% preferred the imaging-based fitting (CI^95%^ 0.02–0.43).

#### 3.4.2. CNC word recognition

**FIGURE 4:**
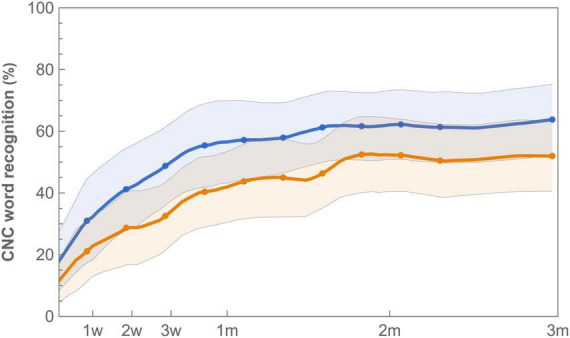
Learning curves for mean CNC word recognition with imaging-based (orange −) and standard (blue −) fitting, which were worn according to a randomization schedule during the first 3 months of CI rehabilitation. Word recognition is defined as the average of test-retest values measured at 65 dB SPL. Error bands indicate 95% confidence intervals.

Mean CNC word recognition scores were higher for standard fitting compared to imaging-based fitting throughout the whole randomization period (Figure 4 and Table 3). This difference was statistically significant when expressed as an AUC [test 3545 (IQR 2102) vs. control 5399 (IQR 2507), *p* = 0.02]. Scores between settings were not significantly different at first fit (*p* = 0.11). Speech recognition after three months was in favor of standard fitting but lost significance after correction [test 46.9% (IQR 34%) vs. control 67.9% (IQR 32.5%), *p* = 0.04]. Additionally, on a group level, subjects showed a steeper learning curve, expressed as average daily improvement, within the first month with the standard fitting [test 0.9% (IQR 0.8%) vs. control 1.3% (IQR 1.1%), *p* = 0.04]. When analyzed for the PP population, a significant difference also occurred for AUC (*p* = 0.03), but not for scores at first fit (*p* = 0.08), after 3 months (*p* = 0.07) or for learning rate (*p* = 0.05). Individual CNC learning curves are presented in Supplementay Figure 2 and show high variability between subjects. For example, the subjects that continued using the imaging-based fitting (EP03 and EP12) obtained higher CNC performance with the imaging-based fitting than with standard fitting, while others (EP01, EP02, EP05, and EP14) scored substantially better with the standard fitting, and another group only showed a minor difference in CNC performance between both settings (EP04, EP09, and EP10).

**TABLE 3:** Word and sentence recognition analyzed by ITT for variables AUC (area under the curve), Begin (first fit), and End (after randomization period).

	Standard fitting (control)			Imaging-based fitting (test)			Difference	
	**Median**	**IQR**	**CI^95%^**	**Median**	**IQR**	**CI^95%^**	**Median**	***p*** **-value**
**CNC word recognition (%)**								
AUC	5,399	2,507	3,762–6,096	3,545	2,102	2,830–4,932	1,854	**0**.02*
Begin	16.0	24.0	0–24	2.25	21.0	0–21	13.75	0.11
End	67.9	32.5	53.1–79.6	46.9	34.0	36.9–70.8	21	0.04*
Learning rate	1.3	1.1	0.9–1.8	0.9	0.8	0.7–1.5	0.5	**0**.04*
**Sentence recognition in quiet (%)**								
AUC	6,887	2,533	5,115–7,558	5,547	3,994	3,857–7,351	1,340	<**0**.01**
End	89.2	15.3	80.2–95.4	79.1	36.6	57.1–93.9	10.1	<**0**.01**
**Sentence recognition in noise (dB SNR)**								
AUC	309	512	64–531	520	697	210–878	−211	**0**.04*
End	0.25	4.9	−3.2 to 1.6	1.9	9.9	−2 to 7.3	−1.65	0.03*

Results are presented as medians with interquartile ranges (IQR), bootstrapped 95% confidence intervals and differences calculated with the Wilcoxon signed-Rank test. Bold font indicates significant difference (with variables Begin and End corrected with Holm-Bonferroni). *p < 0.05, **p < 0.01.

#### 3.4.3. Sentence recognition

**FIGURE 5:**
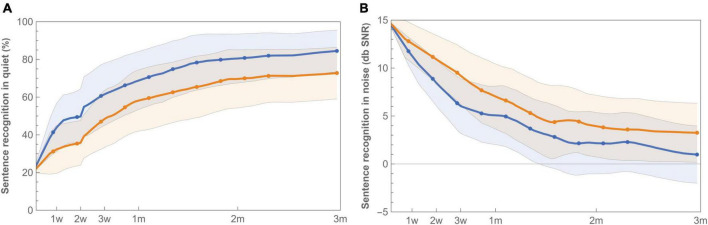
Learning curves for mean sentence recognition in quiet **(A)** and noise **(B)** with imaging-based (orange −) and standard (blue −) fitting, which were worn according to a randomization schedule during the first 3 months of CI rehabilitation. Sentence recognition in quiet is presented as percent correct, while sentence recognition in noise is expressed as the Speech Reception Threshold (SRT), for which a lower value indicates better speech understanding. Error bands indicate 95% confidence intervals.

Sentence recognition was measured both in quiet and in background noise (Figure 5). At the end of randomization, scores with standard fitting were significantly better compared to results with imaging-based fitting in both conditions (Table 3). Final median sentence recognition in quiet was 89.2% (IQR 15.3%) for control and 79.1% (IQR 36.6%) for test (*p* = 0.01). In noise, median scores were 0.25 dB SNR (IQR 4.9 dB SNR) for the standard fitting, and 1.9 dB SNR (IQR 9.9 dB SNR) for the imaging-based fitting, where lower scores indicate better speech understanding. AUCs were also significantly different both in quiet [test 5547 (IQR 3994) vs. control 6887 (IQR 2533), *p* = 0.01] and in noise [test 520 (IQR 697) vs. control 309 (IQR 512), *p* = 0.01]. When analyzed for the PP population, sentence recognition in quiet remained significantly different between settings (End: *p* = 0.01, AUC: *p* = 0.01), while sentence recognition in noise remained significant at the end of randomization (*p* = 0.02), but not for AUC (*p* = 0.05).

#### 3.4.4. Subjective rating

**FIGURE 6:**
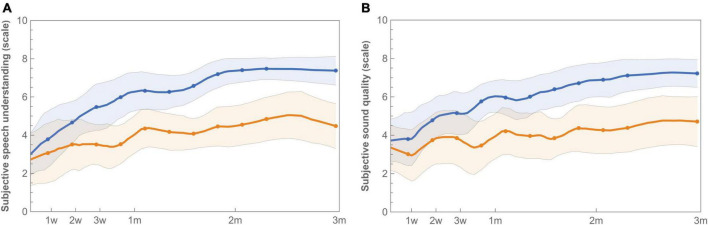
Longitudinal mean satisfaction of speech understanding **(A)** and sound quality **(B)** with imaging-based (orange −) and standard (blue −) fitting. Ratings were performed at each visit using a 10-point satisfaction VAS scale. Error bands indicate 95% confidence intervals.

Satisfaction with speech understanding and overall sound quality for both the imaging-based and standard fitting is shown in Figure 6. Ratings at first fitting were in favor of the standard fitting, but not significantly different (Table 4). After three months, speech understanding for the standard fitting was rated significantly better [test 4.7 (IQR 2.9) vs. control 7.0 (IQR 2.3), *p* = 0.01] which is also reflected in higher AUCs [test 342 (IQR 187) vs. control 559 (IQR 190), *p* = 0.01]. A similar result was found for sound quality, for which the standard fitting was rated as more satisfactory in terms of end result [test 4.5 (IQR 3.1) vs. control 6.9 (IQR 1.9), *p* = 0.01] and AUC [test 327 (IQR 307) vs. control 521 (IQR 178), *p* = 0.01]. When the PP population was analyzed, reported differences between imaging-based and standard fitting remained significant for both speech understanding (End: *p* = 0.01, AUC: *p* = 0.01) and sound quality (End: *p* = 0.03, AUC: *p* = 0.02). Ratings of satisfaction on sound recognition are presented in Supplementary Figure 3.

**TABLE 4:** Preference scales analyzed by ITT for variables AUC (area under the curve), Begin (first fit) and End (after randomization period).

	Standard fitting (control)			Imaging-based fitting (test)			Difference	
	**Median**	**IQR**	**CI^95%^**	**Median**	**IQR**	**CI^95%^**	**Median**	***p*** **-value**
**Rating speech recognition**								
AUC	559	190	481–636	342	187	267–454	217	<**0**.01**
Begin	3.0	2.8	1.5-3.8	1.2	3.0	1.0–4.0	1.8	0.84
End	7.0	2.3	6.6–8.6	4.7	2.9	3.0–5.8	2.3	**0**.01**
**Rating sound quality**								
AUC	521	178	501–631	327	307	288–539	194	**0**.01*
Begin	3.9	1.7	1.6–4.0	3.5	3.1	1.0–6.0	0.4	0.50
End	6.9	1.9	6.9–8.0	4.5	3.1	3.6–6.0	2.4	**0**.01*

Results are presented as medians with interquartile ranges (IQR), bootstrapped 95% confidence intervals and differences calculated with the Wilcoxon signed-Rank test. Bold font indicates significant difference (with variables Begin and End corrected with Holm-Bonferroni). *p < 0.05, **p < 0.01.

### 3.5. Secondary outcomes

#### 3.5.1. Listening effort

**FIGURE 7:**
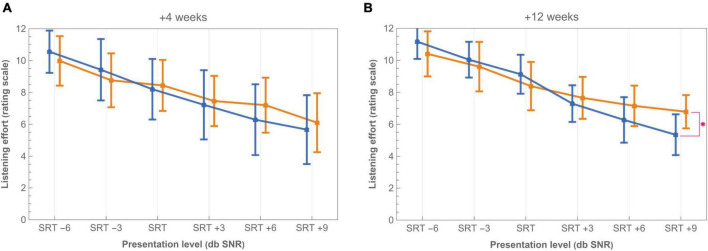
Listening effort in noise with imaging-based (orange −) and standard (blue −) fitting at **(A)** +4 weeks and **(B)** +12 weeks from CI activation. Scores presented are mean ratings on a scale of 0 (no effort) to 12 (extreme effort). Listening conditions are offset on the X-axis to improve readability. Tested levels of SRT –6, SRT –3, SRT, SRT +3, SRT +6, and SRT +9 are expressed relative to the participant’s individual SRT on corresponding conditions (control or test). Asterisk (*) denotes *p* < 0.05 significant difference after applying Holm-Bonferroni correction. Bars indicate 95% confidence intervals.

Listening effort in noise was measured at +4 weeks after CI activation (imaging-based fitting: *N* = 10, standard fitting: *N* = 9) and at +12 weeks (imaging-based fitting: *N* = 12, standard fitting: *N* = 11). Comparing listening effort between the imaging-based and standard fitting revealed no significant differences after correction for multiple comparisons. A *p*-value <0.05 was only found at the most favorable presentation level (SRT + 9 dB SNR) at +12 weeks (Figure 7; *p* = 0.05) in favor of the standard fitting.

#### 3.5.2. Sound quality

**FIGURE 8:**
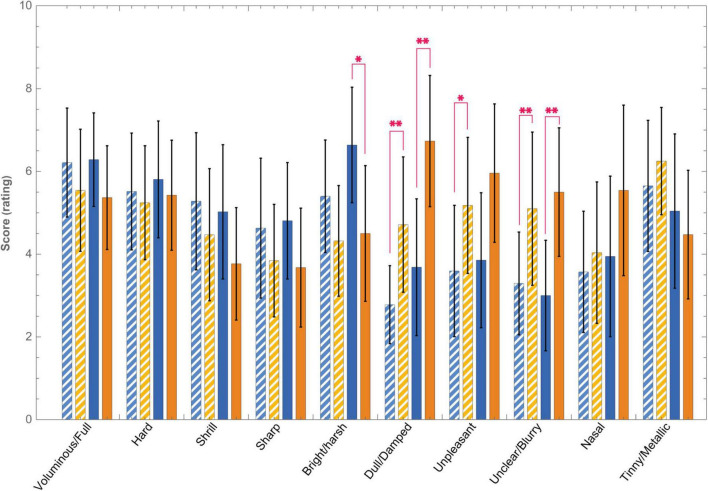
Mean sound quality ratings of 10 features on a scale of 0 (not at all) to 10 (very) with the imaging-based (orange −) and standard (blue −) fitting at +3 weeks (dashed bars) and +12 weeks (solid bars). Asterisk (*) denotes *p* < 0.05 and double asterisk (**) denotes significant difference after applying Holm-Bonferroni correction. Error bars indicate 95% confidence intervals.

Mean ratings of the sound quality questionnaire for the imaging-based and standard fitting are shown in Figure 8 (+3 weeks: *N* = 14, +12 weeks: *N* = 13). The imaging-based fitting was rated as significantly more dull/damped (+3 weeks: *p* = 0.01, +12 weeks: *p* = 0.01) and unclear/blurry (+3 weeks: *p* = 0.01, +12 weeks: *p* = 0.02). Differences on the domains unpleasant (+3 weeks: *p* = 0.04) and bright/harsh (+12 weeks: *p* = 0.04) lost statistical significance after Holm-Bonferroni correction. There were no significant differences for voluminous/full, hard, shrill, sharp, nasal, and tinny/metallic.

### 3.6. Contributors

#### 3.6.1. Aided thresholds

**FIGURE 9:**
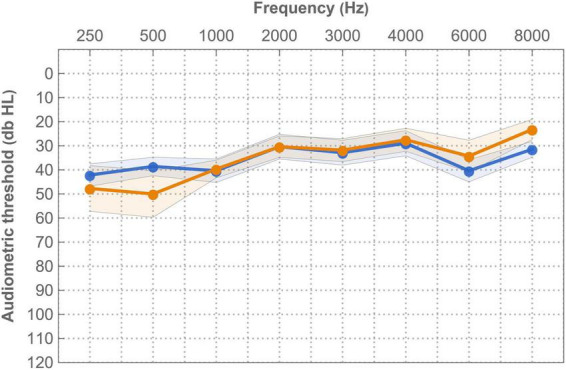
Mean free field (warble) thresholds for the imaging-based (orange −) and standard (blue −) fitting in free field. Measurements were recorded at +1 week. Error bands indicate 95% confidence interval.

Figure 9 shows aided audiometric thresholds with the imaging-based and standard fitting (*N* = 14). Thresholds were significantly different between settings at 500 Hz (more elevated or “worse” with imaging-based fitting) and 8,000 Hz (more elevated with standard fitting). Two subjects had notably elevated thresholds with the imaging-based fitting in the low frequencies: EP01 (250 Hz: 78 dB HL, 500 Hz: 75 dB HL) and EP04 (250 Hz: 75 dB HL, 500 Hz: 80 dB HL) while having better thresholds with the standard fitting (range 30–40 dB HL).

#### 3.6.2. Loudness scaling

**FIGURE 10:**
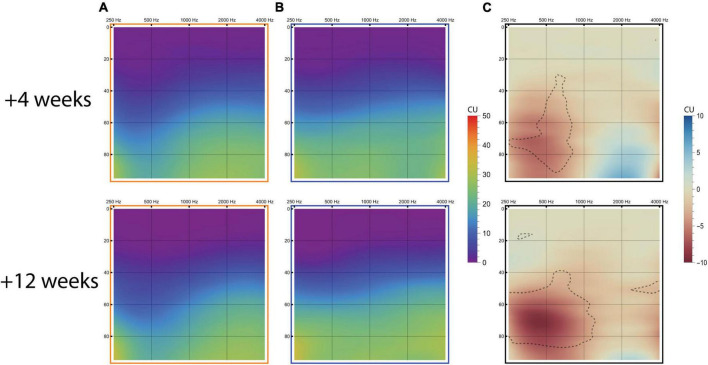
Loudness growth measured with the ACALOS scaling procedure. Results are visualized in loudness audiograms for the imaging-based fitting **(A)**, standard fitting **(B)**, and imaging-based minus standard fitting [**(C)**; red colors resemble more loudness with standard fitting, blue colors indicate more loudness with imaging-based fitting with delineated regions indicating significant differences after Holm-Bonferroni correction]. The graphs display loudness growth with x-axis = frequency (Hz), y-axis = stimulus intensity level (dB HL) and loudness perception (CU) color-coded according to legend.

Mean loudness growth curves for the imaging-based and standard fitting are visualized in Figure 10 (*N* = 12 at both +4 and +12 weeks). Here, ACALOS loudness data were integrated across the frequency spectrum, interpolated in a three-dimensional space and then visualized in a colored graph. These graphs show that differences in loudness growth occurred between settings. Most prominently, within the frequency range 250–500 Hz more loudness growth occurred with the standard fitting, as illustrated by the red areas in Figure 10C. In the high frequencies, blue areas denote greater loudness with the imaging-based fitting, which are present at relatively high stimulus levels (>75 dB HL). Table 5 shows AUCs across frequencies and for each frequency separately. These results demonstrate that the standard fitting produced a louder percept, especially at +12 weeks (*p* = 0.01). In particular, AUCs in the low frequencies were significantly higher with the standard fitting (+12 weeks; 250 Hz: *p* = 0.01, 500 Hz: *p* = 0.01).

**TABLE 5:** Loudness growth with the imaging-based and standard fitting, expressed as AUC across frequencies and for each measured frequency separately.

	Standard fitting (control)			Imaging-based fitting (test)			Difference	
	**Median**	**IQR**	**95% CI**	**Median**	**IQR**	**95% CI**	**Median**	***p*** **-value**
**Loudness scaling (+4 weeks)**								
AUC	45,502	18,943	37,476–56,419	42,487	17,658	33,339–50,997	3,015	**0**.03*
AUC 250 Hz	2,529	799	2,292–3,091	2,457	654	2,229–2,833	72	0.11
AUC 500 Hz	2,537	400	2,287–2,686	2,124	577	1,884–2,461	413	0.03*
AUC 1,000 Hz	2,931	317	2,777–3,094	2,807	285	2,617–2,902	124	0.08
AUC 2,000 Hz	2,886	645	2,479–3,124	3,011	521	2,691–3,212	−125	0.26
AUC 4,000 Hz	3,079	727	2,612–3,339	2,881	695	2,504–3,199	198	0.11
**Loudness scaling (+12 weeks)**								
AUC	49,322	13,228	41,158–54,386	42,093	14,044	34,206–48,250	7,229	**0**.01**
AUC 250 Hz	2,853	546	2,556–3,102	2,605	586	2,358–2,938	248	**0**.01**
AUC 500 Hz	2,576	326	2,475–2,801	2,215	425	1,879–2,305	361	0.01**
AUC 1,000 Hz	2,989	523	2,715–3,238	2,675	366	2,559–2,925	314	0.07
AUC 2,000 Hz	3,077	403	2,946–3,349	2,916	579	2,651–3,231	161	0.46
AUC 4,000 Hz	3,200	613	2,898–3,510	3,106	511	2,728–3,239	94	0.08

Results are presented as medians with interquartile ranges (IQR), bootstrapped 95% confidence intervals and differences calculated with the Wilcoxon signed-Rank test. Bold font indicates significant difference after applying Holm-Bonferroni correction. *p < 0.05, **p < 0.01.

#### 3.6.3. Fitting parameters

Programmed maximum comfortable levels (M levels, expressed in CU) were not significantly different between imaging-based and standard fitting at +3 weeks [intervention 162 CU (IQR 74) vs. control 164 (IQR 66), *p* = 0.78] and + 12 weeks [intervention 172 CU (IQR 64) vs. control 172 (IQR 59), *p* = 0.47]. Programmed T levels were also not significantly different at +3 weeks (*p* = 0.96) and +12 weeks (*p* = 0.26). Median programmed levels at the virtual channel at +3 weeks were 145 CU (M level) and 17 CU (T level) with considerable variation across subjects (IQR M level 72, IQR T level 56). In some patients, the virtual channel seemed to cause subjective discomfort (“echoing”). In these cases, M levels were reduced to minimize complaints. In subject EP02 the virtual channel was disabled at +3 weeks after continued issues. Here, frequencies allocated to the virtual channel were added to the most apical channel.

## 4. Discussion

A single-blinded, daily randomized crossover clinical trial was implemented in order to evaluate a new imaging-based CI mapping strategy directly from the start of the rehabilitation process. For a period of 3 months, starting at first fit, subjects crossed over between the experimental fitting focusing on tonotopic alignment and the standard clinical fitting. On a group level, the standard fitting was superior in all outcome measures, including subjective preference, CNC word recognition, and sentence recognition both in quiet and in noise. In contrast, two out of fourteen subjects preferred the imaging-based strategy. These subjects showed superior speech recognition results with this setting compared to the standard frequency fitting. The variability in subject responses to changes in CI frequency fitting shows the potential for individualizing FATs.

### 4.1. Imaging-based fitting

#### 4.1.1. Factors of influence

It was hypothesized that imaging-based fitting would give rise to a steeper learning curve and improve speech recognition. However, current results show that average learning curves for CNC word recognition, as well as sentence recognition in quiet and noise, were superior for the standard fitting, and standard fitting wins the preference for most subjects. Performance with the standard fitting was higher throughout the first 3 months of CI rehabilitation. Also, subjects did not have a higher learning rate with the imaging-based fitting, instead the opposite proved to be true. This pattern was also prominent for self-rated satisfaction with both settings. Positive sound quality features were more attributed to the standard fitting than the imaging-based fitting, which was described as more dull, unpleasant and unclear.

We noted several factors that could have been of importance in explaining the found differences. Primarily, frequency representation with the imaging-based fitting leaned more toward the high instead of the low frequencies compared to the standard fitting. Over the years, researchers have tried to construct frequency importance functions to identify which frequency regions are most important for speech understanding. Although for listeners with normal hearing these functions have been well established in the Speech Intelligibility Index (with peak band importance at 1–4 kHz), less data is available on which acoustic frequencies CI users rely on (Acoustical Society of America, 1997). Bosen and Chatterjee (2016) estimated band importance by considering speech intelligibility with acoustic stimulation in the presence or absence of each band in both normal hearing listeners and CI users. It was found that where normal hearing listeners demonstrated similar band importance functions, indicating that frequency importance was the same across listeners, CI users showed high variability. Specifically, CI users relied heavily on frequency bands within the 0.2–0.4 and 1–2 kHz region. However, interpretation of these results is complicated in CI users since not only the acoustic frequencies presented to the implant are of importance, but also the place of stimulation. It is possible that the reliance on low frequency bands was not primarily due to the spectral information of the acoustic signal, but because stimulation occurred at cochlear regions that were superior in terms of tonotopic contribution to speech perception or neural health. Authors who investigated the effect of increasing the number of electrodes assigned to low frequencies (below 2,600 Hz) found no significant effect on overall speech recognition (McKay and Henshall, 2002; Leigh et al., 2004). Taking previous results into account, it might be the case that there was insufficient low frequency representation with the imaging-based fitting for adequate speech understanding. To scale down this problem, a rule set was applied that assured predefined low frequency bandwidths (section “2.4. Study intervention”). These rules were the result of an arbitrary trade-off between tonotopic alignment and estimated minimum low frequency representation. Possibly, a different trade-off can be made in the future if more is known about band importance functions for individual CI subjects.

Aided detection thresholds and ACALOS loudness scaling results indeed showed that in the imaging-based fitting low frequencies were underrepresented. Aided thresholds were significantly elevated (“worse”) at 500 Hz with the imaging-based fitting compared to standard fitting. In two subjects (EP01 and EP04), thresholds exceeded 75 dB HL at these frequencies despite comparable M levels for both fittings. Accordingly, loudness growth was significantly less with imaging-based fitting compared to standard fitting. These findings might be explained by the wide frequency bandwidths assigned to apical electrodes or the enabling of the virtual channel, which delivered low-frequency information beyond the most apical electrode. Frequency allocation to this virtual channel was within the range of 238–782 Hz (fixed lower bound, median upper bound 646 Hz), normally covered by four physical electrodes in the standard fitting, and thus might have affected low frequency representation. Some patients did not seem to respond well to the virtual channel and reported subjective complaints such as echoing. This was most prominent during fitting, where, despite extensive efforts, an adequate stimulation level of this virtual channel could not be attained due to side-effects experienced by the patient. As the feasibility of the Phantom functionality seems to differ between patients, further research is needed to address its clinical applicability.

Another factor to considered is the difference in the total number of activated electrodes between fittings. On average, the imaging-based fitting map had three electrodes less enabled than the standard fitting map. This might have affected performance, although multiple studies have reported that 8–10 independent channels are sufficient for maximal performance (Friesen et al., 2001; Shannon et al., 2011; Berg et al., 2022). As noted previously, in two subjects specific channels in the imaging-based fitting were temporarily incorrectly programmed. Although these misconfigurations might have affected speech intelligibility, the impact was likely neglectable since frequencies were merely overlapped and not omitted. In addition, these patients reported no specific complaints which could reasonably be related to the incorrect programming.

#### 4.1.2. Findings in other studies

Previous interventional studies focusing on frequency allocation in CI subjects are limited and often had a non-blinded or non-controlled design. In Fu et al. (2002) three experienced Cochlear Nucleus-22 CI users were exposed to 3 months of continuous wearing of an experimental setting in which the mean frequency allocation was shifted 0.9 octaves downwards and the frequency range was reduced (75–5,411 Hz). Speech intelligibility was significantly worse with the experimental setting compared to clinical settings at first instance. After 3 months of habituation, results on some outcome measures improved but were still significantly lower with the shifted frequency map compared to baseline as well as post-experiment measurements with the clinical fitting. In a different study, MED-EL implanted subjects received frequency maps based on estimated insertion angles and were free to choose between maps for a trial period of at least 6 weeks (Grasmeder et al., 2014). Settings included a mapping to the Greenwood function, a compressed map limited to the area containing spiral ganglion cells, and the clinical map. Both experimental maps performed worse than the clinical map. Recently, a pilot study was published which evaluated a software-based anatomy based fitting procedure in subjects with a CI from MED-EL (Di Maro et al., 2022). Here, based on post-operative CT scans, mismatch was minimized within the 950–3,500 Hz region while mismatch at low and high frequencies was tolerated. In this case, speech intelligibility was reported to be improved with the experimental map. Direct comparisons between previous reports and the current study are complicated as there are major differences in methodology. Also, the other studies included experienced CI subjects who already endured long-term familiarization to their standard CI settings. As motivated previously, the current study exposed subjects from start of rehabilitation to both control and test simultaneously to enable equal learning between settings.

#### 4.1.3. Individual preferences

Although the standard fitting was superior in terms of subject preference and speech intelligibility on a group level, individual learning curves (Supplementary Figure 2) demonstrate the large individual variation in fitting preference. In fact, two out of fourteen subjects preferred the imaging-based fitting (EP03 and EP12) and chose to retain this setting after the randomization period. Moreover, these subjects also objectively performed better with regard to word and sentence recognition compared to the standard fitting. The question then arises why these specific individuals throve with the imaging-based fitting and others did not? Insertion depth and corresponding frequency distribution in the imaging-based FAT were not notably different for EP03 and EP12 compared to other individuals. Namely, tonotopic location of the most apical electrode was 725 and 957 Hz for EP03 and EP12 respectively compared to a group median of 852 Hz for the other individuals. Thus, it seems likely that the abilities of the subjects to extract information from both FATs are different. Possibly, differences in FAT preference can be explained by variability in neural health across the cochlea between subjects. Some areas of the cochlea might be less viable to stimulate electrically due to the occurrence of retracting neurites, reduced integrity of spiral ganglion cells and dead regions, a common phenomenon in sensory-neural hearing loss (Pepler et al., 2014). Here, the neural survival pattern in the individual cochlea might have been different between subjects and have altered the success of fitting strategies. For example, a hypothesis might be that in EP03 and EP12, more important spectral information was allocated to cochlear sites with superior neural health in the imaging-based fitting compared to the standard fitting. If dead regions occurred in the basal region of the cochlea, the effects of both fittings are also likely to be different since basal electrodes were often disabled in the imaging-based fitting. Another explanation of individual variability might be the difference in frequency importance functions between CI users, as mentioned previously. Possibly, those subjects that preferred the imaging-based fitting showed a tendency to extract information more efficiently from higher frequencies, which are represented better in the imaging-based fitting. Other factors have also been considered, such as insertion depth, degree of frequency-to-place mismatch, duration of deafness, etiology, but no clear factors could be identified that explained FAT preference.

### 4.2. Study design

In this study, a trial design was proposed that uses daily crossover randomization as a strategy to prevent first-order carryover effects due to initial brain plasticity related to any CI fitting strategy that is given first. This introduced a new concept for CI users: having to adapt to two CI fittings at the same time during rehabilitation. In a separate manuscript, we will present further data describing long-term learning curves of our study subjects and discuss how learning with two fittings simultaneously might affect performance and underlying mechanisms of brain plasticity. Here, it is also addressed whether absolute learning speed of either map was affected due to the exposure being distributed over twice the amount of time. A prerequisite for successful implementation of daily randomization is that subjects comply to the wearing schedule that allocates between fittings on a daily basis. Here, compliance was monitored by comparing self-reported wearing time of both fittings with predefined cut-off points (Lambriks et al., 2020). Due to violation of compliance, one subject was removed from the PP population. Although the implemented cut-off points can be considered arbitrary, the median compliance difference between intended and actual wearing of the imaging-based fitting was low (2.4%). This strengthens the feasibility of the study design to be used in future CI studies.

### 4.3. Limitations

In general, this study had a high level of compliance and the pre-published protocol was executed according to plan. A lower number of subjects was recruited due to circumstances (amongst other COVID-19). Significant results on most outcome measures were obtained nonetheless. Several other limitations are notable, which can possibly be taken into account in future studies. For example, accurate localization of cochlear landmarks occasionally was challenging due to limited image resolution of preoperative CT scans. This drives the prerequisite of MRI administration instead of CT. Additionally, the use of a virtual channel in the imaging-based fitting might have been a confounding factor. The virtual channel was not well tolerated in some subjects while encompassing a wide frequency range. This might have evoked suboptimal representation of low frequency information. Another limitation of this study is that compliance procedures were not checked with objective datalogging. As this functionality was not available in the fitting software at the time of this study, compliance with the wearing schedule was checked by subjects noting their daily wearing with both fittings in a diary. The impact of this limitation is therefore likely to be limited.

### 4.4. Future research

Although the imaging-based fitting procedure evaluated in this study was not optimal for most subjects, individual variability in outcomes underline the potential for experiments altering frequency allocation in CI subjects. In the future, different trade-offs between tonotopic alignment and low frequency representation should be evaluated. Also, the functionality of the virtual channel should be further explored, as it might have negatively affected speech outcomes in the current study. In general, future studies should either evaluate different FATs that might provide benefit for most subjects, or describe methods that will aid individualized frequency fitting. Here, it is key to determine parameters that might be able to identify the optimal FAT for each individual patient. One of the key factors of interest is neural health, as any mapping procedure is likely to be influenced by differences in neural survival along the cochlea. Currently, there are no validated methods to measure neural health *in vivo*. Ideally, future research will lead to the development of tools that enable individualized frequency fitting in the clinic. The study design introduced in this study can be implemented in future studies as an alternative to traditional set-ups to prevent issues with carryover effects and limited sample sizes.

### 4.5. Conclusion

A standard frequency allocation table obtained subject preference and showed significantly better speech intelligibility compared to an imaging-based CI fitting strategy pursuing tonotopic alignment in the majority of subjects, in contrast to our expectations. An unexpected high individual variability in outcomes however shows the potential for individualized frequency fitting. Concurrently, a novel trial design based on daily randomization was implemented which proved to be a suitable method for evaluation of cochlear implant interventions.

## Data availability statement

The original contributions presented in the study are publicly available. This data can be found here: https://github.com/LarsLambriks/Data-ELEPHANT-study.

## Ethics statement

The studies involving human participants were reviewed and approved by the Ethics Committee of the Maastricht University Medical Center. The patients/participants provided their written informed consent to participate in this study.

## Author contributions

LL, MH, ED, and JD were involved in protocol design, execution, coordination, and analysis of the study. MJ provided statistical support. JC and KH were involved in protocol design of the study. JH and KH provided support as a medical doctor. EG was involved in protocol design and coordination of the study. All authors contributed to the manuscript revision, read, and approved the submitted version.

## Supplementary material

Supplementary Figure 1 Median allocated frequency distribution of imaging-based (orange −) and standard (blue −) fitting showing lower frequency bounds for each electrode. Median tonotopic calculated frequencies are represented in green. Error bands indicate first and third quartile for the imaging-based fitting (standard fitting is equal for all subjects). V, virtual channel (enabled in imaging-based fitting).

Supplementary Figure 2 Individual learning curves for CNC word recognition with the imaging-based (orange −) and standard (blue −) fitting. Word recognition is defined as the average of test-retest values measured at 65 dB SPL.

Supplementary Figure 3 Longitudinal mean satisfaction of sound recognition (discriminate between sounds from the environment) with imaging-based (orange −) and standard (blue −) fitting. Ratings were performed at each visit using a 10-point satisfaction VAS scale. Error bands indicate 95% confidence intervals.
