# Supplementary material for: Imaging-based frequency mapping for cochlear implants – Evaluated using a daily randomized controlled trial
Source: Front Neurosci. 2023 Apr 13;17:1119933. doi: 10.3389/fnins.2023.1119933 (PMC10133468; doi:10.3389/fnins.2023.1119933)
Supplement: Supplementary file 1 [file Data_Sheet_1.docx]

Supplementary material

**Supplementa**ry **Table 1.** Compliance to wearing schedule in ITT population.

| Subject | N days randomization period | Intended wearing imaging-based fitting | Actual wearing imaging-based fitting | Compliance difference |
| --- | --- | --- | --- | --- |
| EP01 | 81 | 48.7 | 49.0 | 0.3 |
| EP02 | 91 | 55.0 | 39.5 | 15.5 |
| EP03 | 89 | 56.4 | 52.4 | 4.0 |
| EP04 | 82 | 47.5 | 57.1 | 9.6 |
| EP05 | 97 | 52.6 | 51.8 | 0.8 |
| EP06 | 84 | 48.8 | 44.5 | 4.3 |
| EP07 | 75 | 52.4 | 26.6 | 25.8* |
| EP08 | 83 | 43.8 | 45.5 | 1.7 |
| EP09 | 94 | 46.9 | 46.5 | 0.4 |
| EP10 | 84 | 50.0 | 45.1 | 4.9 |
| EP11 | 92 | 50.6 | 51.5 | 0.9 |
| EP12 | 85 | 48.2 | 45.2 | 3.0 |
| EP13 | 92 | 45.9 | 47.0 | 1.1 |
| EP14 | 84 | 51.1 | 51.9 | 0.8 |
| Median | 84.5 | 49.4 | 46.8 | 2.4 |
| IQR | 8.5 | 4.4 | 6.6 | 3.9 |

*Removed from PP.

**Supplementary Table 2.** Individual frequency distribution of imaging-based fitting showing lower frequency bounds for each electrode.

|  |  | EL16 | EL15 | EL14 | EL13 | EL12 | EL11 | EL10 | EL9 | EL8 | EL7 | EL6 | EL5 | EL4 | EL3 | EL2 | EL1 | V |
| --- | --- | --- | --- | --- | --- | --- | --- | --- | --- | --- | --- | --- | --- | --- | --- | --- | --- | --- |
| Imaging-based frequency allocation (Hz) | EP01 | - | - | 8598 | 7239 | 6219 | 5200 | 4384 | 3704 | 3161 | 2549 | 2141 | 1801 | 1393 | 1054 | 850 | 646 | 238 |
|  | EP02 | - | - | - | 8598 | 8394 | 6831 | 5675 | 4996 | 4248 | 3636 | 3093 | 2617 | 2005 | 1529 | 986 | 646 | 238 |
|  | EP03 | - | - | - | - | - | 8598 | 7239 | 4996 | 4520 | 3364 | 2753 | 2073 | 1665 | 1257 | 986 | 714 | 238 |
|  | EP04 | - | - | 8598 | 8326 | 7307 | 6083 | 4996 | 4248 | 3568 | 2889 | 2277 | 1801 | 1393 | 1054 | 782 | 646 | 238 |
|  | EP05 | - | 8598 | 8530 | 7103 | 5879 | 5268 | 4656 | 4044 | 3296 | 2821 | 2141 | 1869 | 1393 | 1054 | 850 | 578 | 238 |
|  | EP06 | - | - | 8598 | 8326 | 6976 | 6287 | 5607 | 4996 | 4248 | 3704 | 3093 | 2277 | 1801 | 1189 | 986 | 782 | 238 |
|  | EP07 | - | - | 8598 | 7103 | 5879 | 5268 | 4588 | 4044 | 3296 | 2821 | 2141 | 1869 | 1393 | 1054 | 850 | 578 | 238 |
|  | EP08 | - | 8598 | 7850 | 6831 | 6151 | 5404 | 4656 | 4044 | 3500 | 2957 | 2617 | 2141 | 1665 | 1393 | 986 | 646 | 238 |
|  | EP09 | - | - | 8598 | 7307 | 6491 | 5404 | 4656 | 3704 | 2957 | 2549 | 2073 | 1801 | 1529 | 1325 | 986 | 646 | 238 |
|  | EP10 | - | - | - | - | 8598 | 7986 | 6763 | 6015 | 5064 | 3976 | 3364 | 2685 | 2005 | 1529 | 986 | 646 | 238 |
|  | EP11 | - | - | - | - | 8598 | 7850 | 6355 | 5471 | 4724 | 3976 | 3364 | 2685 | 2005 | 1529 | 986 | 646 | 238 |
|  | EP12 | - | - | - | 8598 | 8394 | 7443 | 6559 | 5336 | 4656 | 3976 | 3364 | 2685 | 2005 | 1529 | 986 | 646 | 238 |
|  | EP13 | - | - | 8598 | 8462 | 7511 | 6627 | 5539 | 4724 | 4112 | 3432 | 2821 | 2413 | 2005 | 1529 | 986 | 646 | 238 |
|  | EP14 | - | 8598 | 7850 | 6559 | 5811 | 5200 | 4384 | 3772 | 3296 | 2821 | 2345 | 2005 | 1665 | 1393 | 986 | 646 | 238 |

Misconfigurations as explained in section 3.2 are not shown. V = Virtual channel.

**Supplementary Table 3.** Median frequency distribution of imaging-based and standard fitting across the electrode array.

|  |  | EL16 | EL15 | EL14 | EL13 | EL12 | EL11 | EL10 | EL9 | EL8 | EL7 | EL6 | EL5 | EL4 | EL3 | EL2 | EL1 | V |
| --- | --- | --- | --- | --- | --- | --- | --- | --- | --- | --- | --- | --- | --- | --- | --- | --- | --- | --- |
| Standard frequency allocation (Hz) |  | 8054 | 4248 | 3568 | 3025 | 2549 | 2141 | 1801 | 1529 | 1257 | 1054 | 918 | 782 | 646 | 578 | 442 | 238 | - |
| Mismatch default fitting (octave) | Median | 0,84 | 1,52 | 1,47 | 1,47 | 1,48 | 1,54 | 1,55 | 1,56 | 1,61 | 1,59 | 1,54 | 1,45 | 1,38 | 1,21 | 1,32 | 1,84 | - |
|  | IQR | 0,37 | 0,55 | 0,56 | 0,47 | 0,46 | 0,49 | 0,45 | 0,32 | 0,46 | 0,39 | 0,54 | 0,43 | 0,58 | 0,6 | 0,53 | 0,57 | - |
| Imaging-based frequency allocation (Hz) | Median | - | 8598 | 8598 | 7307 | 6976 | 6185 | 5267 | 4486 | 3840 | 3160 | 2685 | 2107 | 1665 | 1359 | 986 | 646 | 238 |
|  | IQR | - | 0 | 238 | 1325 | 2311 | 2175 | 1699 | 952 | 1224 | 883 | 952 | 748 | 612 | 475 | 136 | 0 | 0 |
|  | N | 0 | 3 | 9 | 11 | 13 | 14 | 14 | 14 | 14 | 14 | 14 | 14 | 14 | 14 | 14 | 14 | 14 |
| Mismatch imaging-based fitting (octave) | Median | - | 0,17 | 0 | 0 | 0 | 0 | 0 | 0 | 0 | 0 | -0,01 | -0,01 | 0,01 | 0,03 | 0,16 | 0,4 | N.A. |
|  | IQR | - | 0,15 | 0,17 | 0,08 | 0,01 | 0,01 | 0,01 | 0,02 | 0,01 | 0,02 | 0,02 | 0,04 | 0,06 | 0,09 | 0,31 | 0,58 | N.A. |
|  | N | 0 | 3 | 9 | 11 | 13 | 14 | 14 | 14 | 14 | 14 | 14 | 14 | 14 | 14 | 14 | 14 | 14 |

Mismatch is calculated in octaves for each FAT compared to Greenwood position for each electrode. V = Virtual channel. IQR=Interquartile Range.

**Supplemental Table 4**. Spectral ripple discrimination with the imaging-based and standard fitting. Values from the last six reversals of the SMRT test were averaged to give a threshold (in ripples per octave).

|  |  | Standard fitting (control) | | | Imaging-based fitting (test) | | | Difference | |
| --- | --- | --- | --- | --- | --- | --- | --- | --- | --- |
|  |  | Median | IQR | 95% CI | Median | IQR | 95% CI | Median | P value |
| Spectral ripple discrimination (ripples per octave) | | | | | | | | | |
| +4 weeks |  | 2.15 | 2.82 | 1.41-3.43 | 1.60 | 1.03 | 1.10-2.88 | 0.55 | 0.30 |
| +12 weeks |  | 1.88 | 1.13 | 1.42-3.22 | 1.47 | 1.08 | 1.00-3.02 | 0.41 | 0.23 |

The procedure was administered two times and means of test-retest were recorded. Results are presented as medians with interquartile ranges (IQR), bootstrapped 95% confidence intervals and differences calculated with the Wilcoxon signed-Rank test. No significant differences were found between settings.
