# Supplementary figures and images for: Imaging-based frequency mapping for cochlear implants – Evaluated using a daily randomized controlled trial
Source: Front Neurosci. 2023 Apr 13;17:1119933. doi: 10.3389/fnins.2023.1119933 (PMC10133468; doi:10.3389/fnins.2023.1119933)

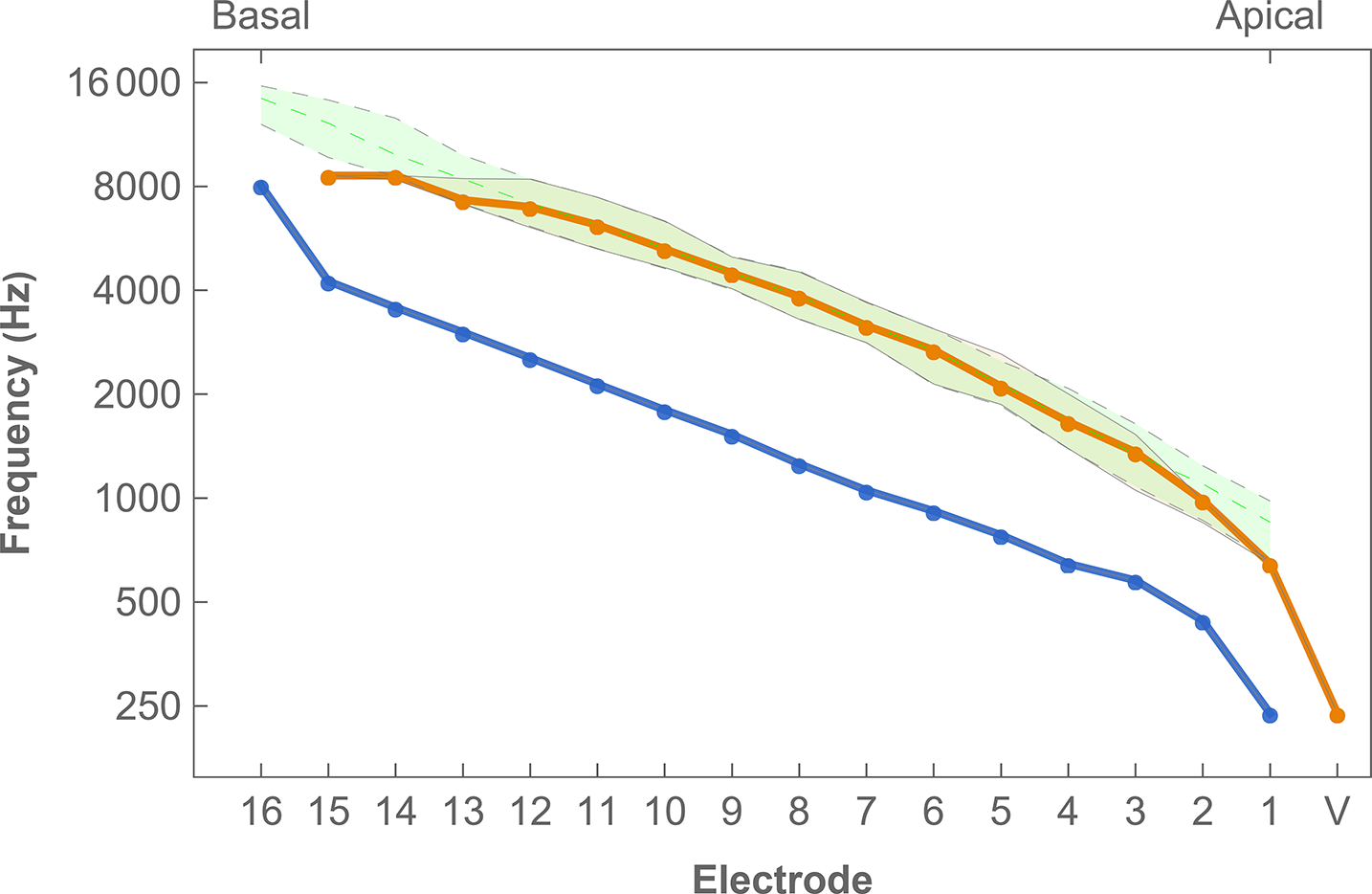

Supplement: Supplementary Figure 1 — Median allocated frequency distribution of imaging-based (orange −) and standard (blue −) fitting showing lower frequency bounds for each electrode. Median tonotopic calculated frequencies are represented in green. Error bands indicate first and third quartile for the imaging-based fitting (standard fitting is equal for all subjects). V, virtual channel (enabled in imaging-based fitting). [file Image_1.TIFF]

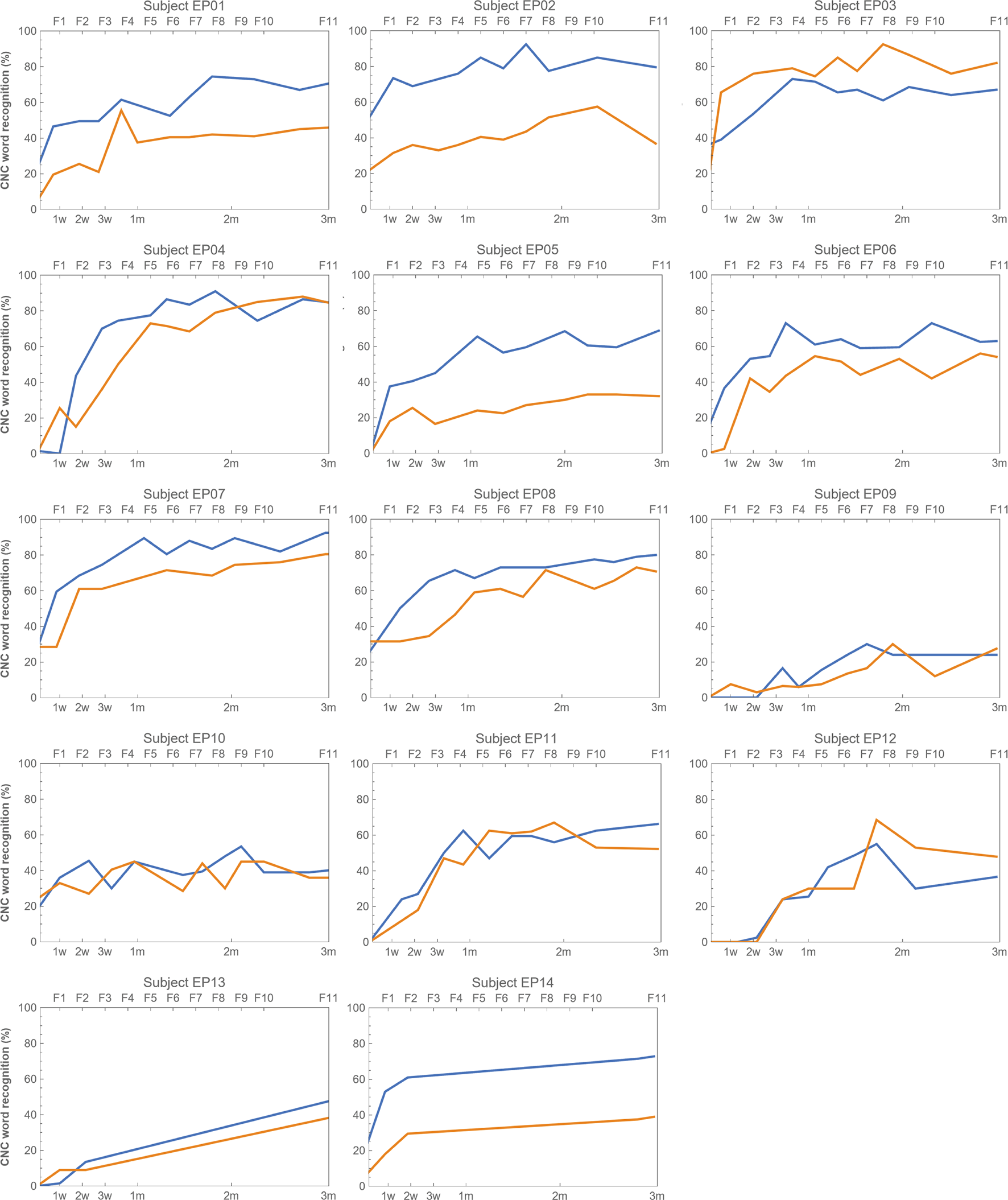

Supplement: Supplementary Figure 2 — Individual learning curves for CNC word recognition with the imaging-based (orange −) and standard (blue −) fitting. Word recognition is defined as the average of test-retest values measured at 65 dB SPL. [file Image_2.TIF]

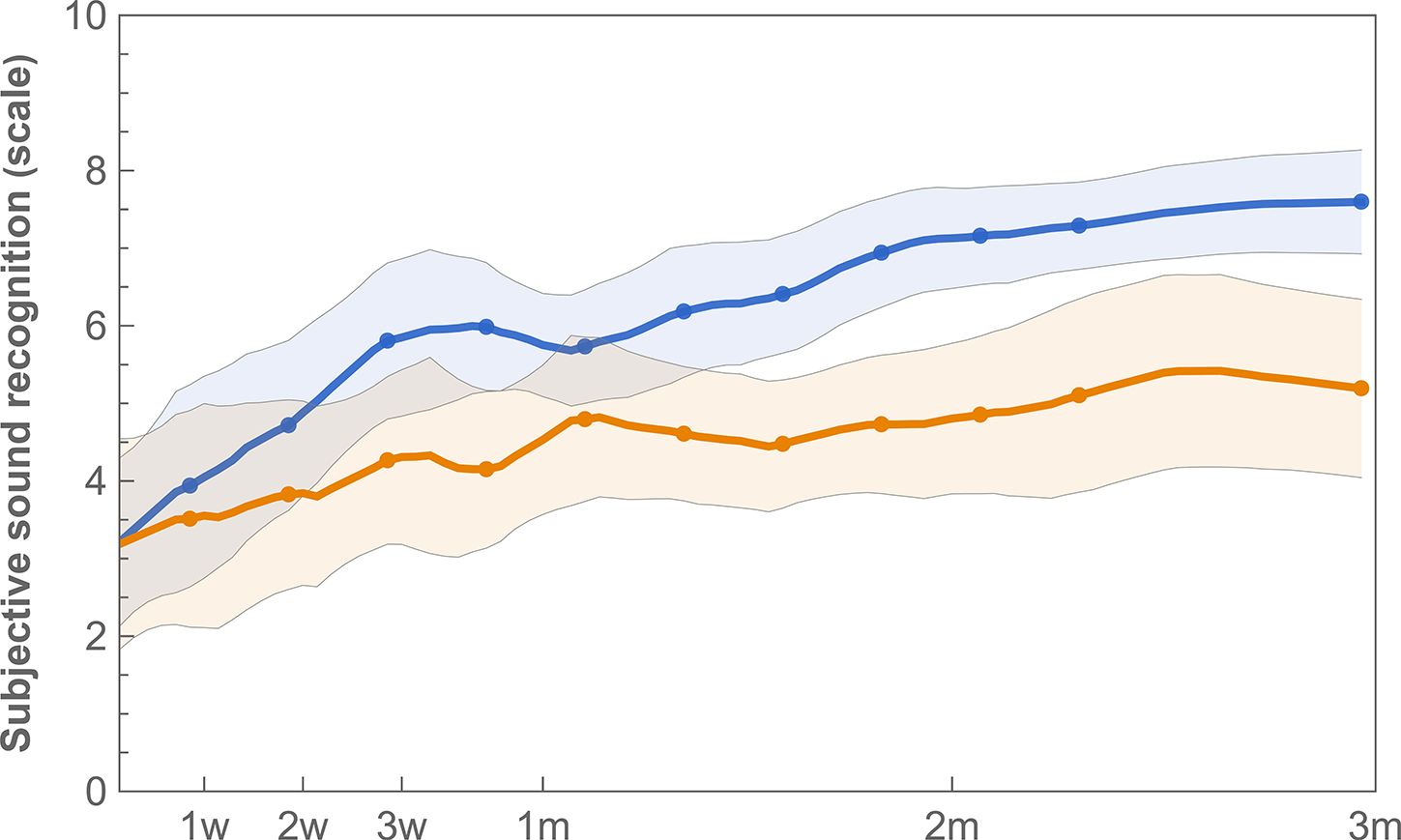

Supplement: Supplementary Figure 3 — Longitudinal mean satisfaction of sound recognition (discriminate between sounds from the environment) with imaging-based (orange −) and standard (blue −) fitting. Ratings were performed at each visit using a 10-point satisfaction VAS scale. Error bands indicate 95% confidence intervals. [file Image_3.TIFF]
